# Digital and competing information sources: Impact on environmental concern and prospects for international policy cooperation

**DOI:** 10.1007/s10368-021-00503-8

**Published:** 2021-08-18

**Authors:** Vladimir Udalov, Paul J. J. Welfens

**Affiliations:** 1 University of Wuppertal, Gaußstraße 20, 42119 Wuppertal, Germany; 2 EIIW, Rainer-Gruenter-Str. 21, Campus Freudenberg, 42119 Wuppertal, Germany

**Keywords:** Environmental concern, World value survey, Climate policy, Information technologies, International economics

## Abstract

The environmental concern of people in industrialized and developing countries is analyzed. Using the 2010–2014 wave of the World Value Survey (WVS), the main purpose of our analysis is to investigate the effect of different information sources on the affective, conative and behavioral components of the environmental concern of people in the developed and developing countries. As independent variables, we use a set of economic data as well as information-related variables, including the internet, mobile phones, TV, radio and newspapers. The digital variables of the internet and mobile phones turn out to have a highly significant impact on environmental concern so that digital modernization of countries should have pro-environmental impacts as a side effect of internet and mobile phone services expansion. With the developing countries catching-up vis-à-vis the OECD countries in the field of mobile phone density and internet density, respectively, one may expect better prospects for cooperation between developed and developing countries since attitudes/the environmental concern of people in developed and developing countries will become more similar. For international green cooperation and climate change policy progress, the new findings presented herein are crucial.

## Introduction

Many industrialized as well as newly industrialized countries (NICs) have adopted policy initiatives aimed at improving the quality of the environment or at fostering some form of green growth and the development of new, CO2-light, technologies which could help to achieve the stabilization of the climate and to avoid global warming, respectively. Middle-of-the-road, or centrist, parties as well as special issue parties with a strategic focus on environmental issues – for example the Green parties in many European Union (EU) countries – represent the interests of voters in the field of environmental quality and combatting global warming; in the elections for the European Parliament in 2019, the Green Party in Germany, in particular, increased its voting share and the exit polls of the German expert electoral analysis group *Forschungsgruppe Wahlen* indicated that a large share of voters hold the view that the EU should become more active in climate change policy (Forschungsgruppe Wahlen 2019). This seems to suggest broader individual support for sustainability policy in Germany, for example, however there is no clear picture at an international level as to the extent to which individuals in many countries in the North and the South of the world economy support pro-sustainability policy and ambitious climate policy as emphasized by the United Nations (UN). As regards macro perspectives on climate change, it is not obvious to many that there is a double dividend possible if CO2 pricing-related revenues in OECD countries are used for fiscal recycling to the households through effectively lower income tax rates. Green progress and high per capita income could still be a trade-off setting so that individuals’ perceptions and attitudes about the environment and economic well-being should matter – as should the communication channels available. In the published report of the IPCC (2021), it has clearly been argued that the natural science base findings further underline the analysis of previous IPCC research and even suggest that the 1.5 degrees limit for global warming might already become a reality by 2030, earlier than was anticipated in previous reports. From this perspective, the attitude of people with respect to sustainability and climate change issues in different countries are indeed of critical relevance for international cooperation and policy progress; and the relevance of alternative modes of diffusion of crucial information seem to be quite important in this context.

The UN COP25 conference in Madrid in 2019 was extended beyond the initial program as extra days were required for continuing negotiations which, ultimately, brought little in the way of political success, mainly due to the fact that conferences with over 190 participants are complex political events where high transaction costs for negotiations and free rider problems in the provision of the global public goods of CO2 mitigation and climate policy, respectively, make achieving progress towards attaining a global consensus quite difficult.

Surprisingly, there is not much knowledge about the environmental concern of individuals nor their willingness to pay for environmental improvement—and the role of relevant information sources, including internet access. Such knowledge would be important not only from an international comparative perspective, but could also be a basis – combined with other relevant knowledge – for a better understanding of opportunities for international environmental and economic policy cooperation; not only are the sources of information regarding environmental concern crucial but also the role of economic and psychological control variables.

Macroeconomic policy approaches could be environmentally-friendly and indeed could support climate-friendly structural change: Looking at a combination of higher CO2 taxes, tax recycling through lower income tax rates to a large extent (say 80%) plus the remaining revenue devoted to higher R&D promotion by government, could be an adequate approach for an optimal green tax reform; the higher CO2 tax brings lower CO2 emissions and thus reduces negative external effects from the use of fossil fuel energy sources, the reduction of income tax rates reduces the negative welfare effects from CO2 taxation and the rise of green R&D promotion contributes to higher positive external effects from higher innovation dynamics (Welfens 2019). These macroeconomic perspectives suggest possible welfare-enhancing perspectives of green growth policy, but there is double caveat: Firstly, government would really have to consider and implement such a new policy approach in a consistent way and, secondly, the attitudes and the behavior of individuals (read voters) should be such that there is indeed broad support for policymakers eager to modernize economic policy in a climate-friendly way or new policy measures that generally enhance sustainability. Do individuals in leading industrialized countries and developing countries really support green growth policies? To what extent are the individuals’ attitudes and views different in a North–South comparative perspective so that – beyond the impact of per capita income – a comprehensive international policy cooperation is difficult to achieve? Here, the World Value Survey results have to be considered and in particular the channels by which “environmental concern” are shaped. As regards a European Green Deal, as envisaged by the European Commission (2019), it is also crucial to have a basic understanding as to the extent to which attitudes of people in EU countries oriented towards supporting measures aimed at climate protection and how potential trade-off relations are considered in society. The same also applies to other countries in the world economy, but thus far it is still relatively unclear just how attitudes are shaped through various information channels and other influences.

The following analysis aims at closing this research gap by taking a look at survey results from 46 industrialized and developing countries in 2010–14, where not only alternative information sources – traditional as well as digital—for both high-income countries and low-income countries are considered but also a group of control variables. As regards differences between high-income and low-income countries, it is clear that there is a considerable digital structural gap between the two groups – relevant for internet information and digital communication aspects; however, over time the digital mobile gap between developed and developing is expected to be closed fairly quickly (ITU 2015, 2018) and certainly faster than the per capita income gap. While different attitudes, priorities and green activity levels are often making cooperation between developed and developing countries in environmental policy difficult, one should consider the medium and long-run prospects for behavioral green convergence across countries as well – if one would know more about the underlying variables, it would be easier to adopt more efficient and consistent international cooperation approaches in environmental policy.

In Environmental Economics there is a wide range of survey studies for EU countries and the US; as regards survey results on the individual’s willingness to pay for climate protection, there is a broad view that answers from surveys will be biased since climate protection and many other fields of environmental policy stand for a national/international public good. If one carefully considers the survey design necessary to avoid biased results, one will receive, in the field of willingness to pay for reducing greenhouse gas (GHG) emissions, valid important results which suggest in the case of Germany that environmental concern so far is crucial for an influential minority from the upper strata of society (Löschel et al. 2010). A majority of those German respondents surveyed in a ZEW study had zero willingness to pay for reduced GHG emissions, the minority that showed some significant willingness to pay was characterized by high per capita incomes and rather high levels of education plus a party preference in favor of the Green Party (an ecological party in Germany). Willingness to pay in the US has been studied by Kotchen et al. (2013) who found that household surveys showed a positive willingness to pay for reducing domestic greenhouse gas emissions – with higher education raising willingness to pay, while older individuals were found to have a lower willingness to pay for a carbon tax and GHG regulation; a higher income raised the willingness to pay for both policy instruments mentioned. In a broader perspective, the key international challenge is sustainability and resource efficiency (Bleischwitz et al. 2009; Welfens et al. 2015).

Wagner (2016) has shown for the case of the US that consumers who are environmentally-conscious are characterized by lower gasoline net price and excise tax elasticities (net means: tax-exclusive). As regards price signals this implies that this group is less sensitive to changes in green taxes and prices than less environmentally-conscious households. This not only indicates support for the role of heterogenous environmental preferences, but also suggests that the diffusion of environmental-consciousness could, paradoxically, reduce the environmental impact of green taxes – this link stands for a new analytical challenge that might become relevant in the context of climate change policy. It is not clear that a similar phenomenon of heterogenous environmental preferences also matters in Asia or the EU; given the new “green” emphasis of the European Union’s Von der Leyen Commission, this question could play a crucial role for an effective EU climate change policy – climate change policy will also become a new serious challenge for the G20 group (Welfens 2019).

The following study makes use of the 2010–2014 wave of the World Value Survey, analyzing more than 66,000 survey responses across 46 countries. A key idea in the subsequent empirical analysis is to make a distinction between affective (the individual considers environmental issues as a pressing problem which is reflected in the personal attitude), conative (environmental protection is said to be preferred over growth so that a well-reflected interest in a clean environment is indicated) and pro-environmental action (the individual has donated money for the improvement of the environment) as three different dimensions of environmental concern; for each of the three pillars of environmental concern there is a need to use information, not least information relevant for green topics and issues. So, which of the many alternative information sources (radio, newspaper, internet etc.) is relevant for the three degrees of environmental concern where the weakest level of concern is represented by attitude, the second weakest by conative perspective and the strongest by pro-environmental action?

The basic finding of the subsequent probit regression analysis is that with respect to alternative information sources the internet is significant for all three types of environmental concern – for the version of the preferred model with country dummy. When it comes to action, all information sources are significant except for conversations with other people. Among the economic control variables considered, the income variable and the education variable, as well as socio-ecological attitudes, are relevant for all three green levels of commitment; plus the internet penetration intensity (country indicator) for “Green Intention” and “Environmental Activity”. The findings differ partly between the high-income group and the low-income group as will be explained subsequently.

The setup of the analysis is such that in Sect. (2) there is a brief descriptive part and a brief presentation of the analytical framework, while Sect. (3) gives selected empirical findings. Section (4) presents conclusions and policy implications.

## Literature and analytical framework

In markets with network effects – e.g. telecommunications markets but also some two-sided markets (e.g. credit cards) – the increasing demand of users will raise the marginal benefits for early users so that there is an endogenous rise of market demand and the equilibrium quantity, respectively. In a social context – with the new equilibrium quantity defining a certain standard level to be achieved from younger cohorts of demanders as well – a certain benchmark, i.e. a new social norm, could be established. Beyond these considerations, one may also be interested in the psychological links between pro-environmental intentions and environmental actions; how strong are these links, how can they be influenced and how are policy options thereby affected. It seems fairly obvious that those with pro-environmental actions will have underlying pro-environmental intentions. However, many individuals with green intentions might exhibit little pro-environmental activity and generally one may assume that relevant aspects for the respective link will be found when looking at information, knowledge, direct individual costs and private benefits versus social benefits.

To some extent one will have to raise the question of how pro-environmental standards are established in various societies. Looking at a broad spectrum of publications on environmental and recycling behavior, the meta studies of Hornik et al. (1995) and Visschers et al. (2009) have identified a range of drivers and barriers to desired behavior. Saphores et al. (2012) have argued that certain variables such as income, gender and age are not reliable and effective drivers of recycling. It is interesting that the findings of Darby and Obara (2005) show that in low-income households the recycling intensity is rather low, however a longer use phase is observed – namely, compared to high-income households. Knowledge, habits, certain economic factors and personal attitudes towards the respective subjects as well as existing personal and dominant social norms are found to have an influence on action and behavior, respectively. One might find high income-low income environmental attitude differences not only within countries but across countries as well.

In a broad international context, it would be useful to consider the findings from international surveys that allow to build an environmental action variable and a green attitude variable as a basis for empirical analysis on the relative role of internal factors (e.g. attitude) and external factors (e.g. economic control factors) on environmental action. Thus, looking at regional findings in a broader international context should be quite useful. The subsequent analysis makes a new contribution in the sense that new survey data on green attitudes and pro-environmental behavior are analyzed and the empirical section then also takes a set of adequate control variables into account. As regards the set of more than 40 industrialized and newly industrialized/developing countries covered, one may emphasize a certain caveat, namely, that in some relatively poor countries, autocratic government or specific legislation rule out specific pro-environmental actions that otherwise might easily be found in western OECD countries, Japan or in certain developing countries with particular democratic structures. Looking at various information sources in the international survey to be considered subsequently naturally reveals some complementarity as well as substitutability which are sometimes difficult to disentangle. People will rely, for example, on conventional information sources such as newspapers/magazines to some extent, at the same time people will often have internet access as well as mobile phones (again often with internet access). A priori it is clear that in developing countries fixed-line access is rather small so that mobile phones might play a rather large role as an information source – compared to rich OECD countries where people often have fixed-line internet access, but also have mobile internet services.

**Fig. 1:**
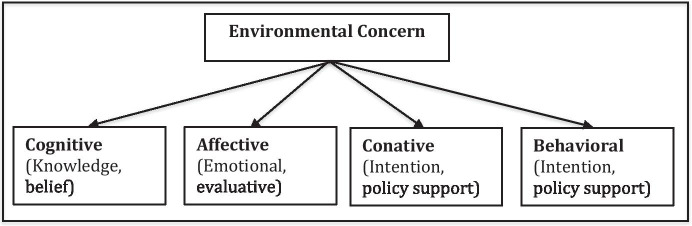
Components of Environmental Concern (Source: Schaffrin (2011))

Individual concern about the environment is a relatively modern dimension of human behavior and individual perception. According to Van Liere and Dunlap (1980), environmental concern can be defined as perceiving environmental problems as serious, supporting efforts by government to protect environmental quality and as engaging in behaviors aimed at improving environmental quality. Schaffrin (2011) decomposes the above definition into cognitive, affective, conative and behavioral components. The cognitive component contains knowledge, beliefs, or norms, whereas the affective component refers to emotive and evaluative individual stages. The conative dimension is an expression of behavioral intention. The behavioral component contains the transmission of the intention into pro-environmental behavior. Pro-environmental behavior can include personal buying behavior, travel behavior, recycling and the use of resources and active participation in a pro-environmental organization (see Fig. 1).

Schaffrin (2011) argues that someone is concerned about the environment, if the person generally accepts (global) environmental problems as being serious and agrees towards environmental policies, shows pro-environmental intentions and takes personal action in order to mitigate environmental pollution. Stern and Dietz (1994) and Stern et al. (1995) investigate the question of why individuals may be concerned about the environment, by identifying three sets of internal factors associated with environmental attitudes. They label them as egoistic, altruistic, and biospheric. Egoistic values are focused on the self and self-oriented goals, altruistic values focus on other people and biospheric values focus on the well-being of living things. Ajzen and Fishbein (1980) argue that the pro-environmental intentions of individuals are an outcome of general beliefs and attitudes. Dunlap and Van Liere (1978) and Dunlap et al. (2000) develop the ‘New Ecological Paradigm’, in which general attitudes about the environment positively affect pro-environmental intentions. The norm-activation model by Schwartz (1977) and Schwartz and Howard (1981) states that pro-environmental behavior occurs in response to personal moral norms that are activated in individuals who believe that particular conditions pose threats to others (AC) and that actions they could initiate may avert those consequences (AR). Based on this, the value-belief-norm theory by Stern and Dietz (1994), Stern et al. (1995), Stern et al. (1999) and Stern (2000) assumes a dominant role of values and norms for pro-environmental intentions and behavior. In a more recent study, Udalov et al. (2017) show that both individual energy efficiency investments and daily energy saving activities are mostly driven by environmental motivations. Yushkova and Feng (2017) show that recycling activities among German and Chinese students are also positively influenced by environmental concern.

According to Gelissen (2007), there is empirical evidence for significant differences among countries in pro-environmental attitudes and behaviors. This leads to the question about the underlying differences in individuals’ perceptions and concerns across countries where informational aspects plus economic control variables as well as personal attitudes might play a role.

The variations among individuals from different countries can be explained using three main hypotheses that focus primarily on the role of income: a post-material hypothesis, an affluence hypothesis and a globalization hypothesis. The post-material hypothesis, developed by Inglehart (1971, 1997), postulates that people tend to embrace more post-materialistic attitudes when socio-economic security rises. As societies become more affluent, their members are less preoccupied with the economic struggle for survival and are free to pursue post-materialistic goals. Because environmental attitudes can reasonably be regarded as part of a general post-materialistic outlook, environmental concern increases due to the shift from materialism to post-materialism. In order to explain concern for the environment in developing economies, Inglehart (1995) uses the “objective problems, subjective values” (OPSV) hypothesis. The OPSV hypothesis states that concern for the environment in developing countries follows from the necessity to overcome objective local environmental problems. The developed economies express environmental concern for reasons justified by post-materialistic subjective values. In addition, one may argue that the role of natural capital in overall capital in poor countries is relatively higher than in industrialized countries (World Bank 2014) so that one could argue that relative factor endowment will also contribute to individual concern about the environment in relatively poor countries; moreover, looking at a green composite indicator, one can not only find OECD countries among the Top 25 but also several developing countries and NICs (Welfens and Lutz 2012; Welfens et al. 2015). 

The affluence hypothesis treats environmental quality as a normal good, with more demand at higher income levels. Dieckmann and Franzen (1999), Franzen (2003), Franzen and Meyer (2010) as well as Franzen and Vogl (2013) show that environmental concern is closely correlated with the wealth of the nations and per capita income, respectively. These studies reveal that respondents in more wealthy nations tend to have higher environmental concern, indicating that environmental concern is also closely associated with post-materialistic attitudes.

The globalization hypothesis predicts that there should be no relation between economic development levels and environmental concern. Dunlap and York (2008) state that citizens’ concern for the environment is not dependent on national affluence, nor on affluence based post-materialist values. According to Dunlap and Mertig (1995), national affluence is in fact more often negatively rather than positively related to citizen concern for environmental quality. This result implicates that public support for environmental protection is not limited to the wealthy nations of the world but actually represents a global phenomenon. Indeed, previous Pew Research has shown broad public concern around the globe with regard to climate change issues (Pew Research Center 2013).

Besides other factors, for example geographical and temporal effects, environmental concern depends significantly on information since more informed individuals seem to have a higher awareness of climate change risks. Environmental concern requires knowledge and information about the causes and consequences of environmental problems (Nistor 2010). According to Juraitë (2002), environmental deterioration becomes a social problem only if society as a whole, or a subgroup, recognizes the environment as a problem. Dunlap and Jones (2002) emphasize the role of media coverage regarding recognition and the social concern towards global environmental problems. Thus, mass media and the media coverage of environmental issues play a crucial role in increasing environmental awareness.

Different information sources might have different effects on environmental concern. According to Ostman and Parker (1987), newspaper use is more likely to lead to increased audience attention to environmental content, increased levels of environmental awareness and concern. As regards television, an increased use of television tends to discourage relevant environmentally positive behavior. However, Holbert et al. (2010) argue that it is important to focus on different types of television use. The use of television news has a positive influence in creating a greater desire within individuals to recycle, purchase products that are environmentally friendly, and be more energy efficient in their daily routines.

Mass media has moved towards new forms of communication technologies and instruments. According to Nistor (2010), internet use plays a crucial role in enhancing individual’s environmental concern since internet has become a new means for mobilization on environmental issues and changes environmental campaigning. Good (2006, pp. 195–216) postulates that “internet is providing a new source of information and connection with others while decreasing the time available for televisions cultivation of environmental apathy”.

## Empirical strategy

Using the 2010–2014 wave of the World Value Survey (WVS), the main purpose of this paper is to analyze the effect of different information sources on affective, conative and behavioral components of environmental concern definition. According to Schaffrin (2011), the affective component represents an evaluative part of environmental concern where individuals decide whether postulated consequences from environmental problems are good or bad. The conative component is an expression of behavioral intention. The behavioral component contains the transmission of the intention into pro-environmental behavior.

We will consider daily newspaper, TV news, radio news, mobile phone, email, internet and conversations with friends or colleagues as the corresponding information courses.

We formulate the following hypotheses regarding the effect of different information sources on the environmental concern:

- **H1**: Since internet is the broadest and fastest source of information, we assume that internet will have a positive significant effect for all dimensions of environmental concern.
- **H2**: Weak levels of environmental concern are linked to a rather narrow information status; the higher the individual commitment for the environment is, the broader the information basis used will be – a reflection of rational behavior in the sense that in the end green action will certainly require information about the respective problem and potentially also about the alternative views and behavioral tendencies of other people. Unlike other technologies, people in developing countries use mobile phones at almost similar rates as developed countries – developing countries have, as is well-known, a fixed-line telecommunications gap compared to OECD countries. Since individuals in low-income countries often lack fixed-line communication access, one may formulate a third hypothesis that emphasizes particularly the role of mobile phones in low-income countries; we state as the third hypothesis:
- **H3**: Use of mobile phones as an information source will have a major influence on environmental concern in the low-income sub-sample.

In order to empirically investigate these hypotheses, we will run regressions on the total sample in the first step. Due to the large heterogeneity between countries in the full sample, it is appropriate to split the full sample into sub-samples in which countries are more similar. Following the affluence and post-material hypotheses, which postulate that pro-environmental intentions are closely correlated with the wealth of the nations because individuals living in richer countries tend to embrace more post-materialistic attitudes, we will split the sample into high- and low-income sub-samples using the IMF classification. The low-income sub-sample is especially crucial for our third hypothesis.

### Data

We will make use of the 2010–2014 wave of the World Value Survey (WVS) with 66,278 survey responses across 46 countries, 25 of which are classified by the IMF as low-income countries. The WVS is designed to be a representative survey carried out using consistent methodologies across numerous countries, focusing “on changes in the beliefs, values and motivations of people throughout the world”. WVS employs a probabilistic sample method and uses a minimum sample size of 1,000 respondents (Israel and Levinson, 2004).

### Dependent variables

The corresponding dependent variables include affective, conative and behavioral components of environmental concern. Since the affective component represents an evaluative part of environmental concern, individuals’ responses to the following question are used:

- Please indicate which of the following problems you consider as the most serious one for the world as a whole?
  - People living in poverty and need
  - Discrimination against girls and women
  - Poor sanitation and infectious diseases
  - Inadequate education
  - Environmental pollution

The conative component is an expression of behavioral intention and is captured by responses to the question regarding the trade-off between economic growth and environmental protection:

- Protecting the environment should be given priority, even if it causes slower economic growth and some loss of jobs.
- Economic growth and creating jobs should be the top priority, even if the environment suffers to some extent.
- Other answer.

The behavioral component is captured by investigating whether or not the respondent has personally taken actions aimed at helping to fight climate change. We consider responses to the following question:

- Have you given money to an ecological organization during the past two years?

The dependent variables are summarized in the following table (see Table 1):

**Table 1:** Overview of dependent variables

Dependent variables	Type	Description	Min	Max	Mean	SD
Affective Component	Binary 0—1	1 if the respondent considers environmental pollution as the most serious problem for the world as whole, 0 otherwise	0	1	0.14	0.35
Conative component	Binary 0—1	1 if the respondent prefers protecting the environment, even if it causes slower economic growth and some loss of jobs, 0 otherwise	0	1	0.52	0.50
Behavioral Component	Binary 0—1	1 if the respondent has given money to an ecological organization during the past two years, 0 otherwise	0	1	0.12	0.33

### Independent Variables

In order to examine the effect of different information sources on affective, conative and behavioral components of environmental concern, we include the responses to the following question regarding the usage frequency of different information sources into the analysis:

- People learn what is going on in this country and the world from various sources. For each of the following sources, please indicate whether you use it to obtain information daily, weekly, monthly, less than monthly or never:
  - Internet
  - Daily newspaper
  - Printed magazines
  - TV news
  - Radio news
  - Mobile phone
  - Talk with friends or colleagues

In the context of the expansion of modern communication networks and information & communication technology (ICT), respectively, the role of digital information opportunities has been reinforced. With the internet increasingly becoming a preferred source of information, one may assume that powerful digital search and networking technology could also play an increasing role for environmental concerns and “green information”. As regards traditional information sources such as newspapers, magazines, TV or radio, one may point out that these are weak substitutes for the global information source of the internet which is very fast and diversified if users employ search engines and user costs are rather low except for the time invested in the search process. All kinds of information sources mentioned so far could indeed provide environmentally relevant information and news which reinforce the readers’/users’ environmental concern. In newspapers, and part of the internet, the standard wisdom of content provision is “bad news is good news” since such news arouses the interest of users. Looking at the specifics of various information sources, one may point out some particular aspects:

Newspaper information is not focused on environmental issues in a regular way and many newspapers are general information sources where the reader uses the newspaper on a subscription basis; typically, all kinds of topics are covered. Newspapers to some extent have migrated onto the internet, but, with the exception of the New York Times, most quality papers in OECD countries in 2014 had a majority of traditional readers using printed editions.

The use of TV can be a strong visual source of information on environmental topics, while documentaries and other films with a green focus or TV news about major international climate conferences could alert many people. IP-based digital TV programs were standard in OECD countries in 2014 and similar findings hold for radio programs. By contrast, in low-income countries the digital element of TV and radio was often still rather limited.

Radio stations typically differ amongst each other, but again one could argue that entertainment (e.g. music programs) dominates the radio services consumption of private households in most countries; radio music listeners might represent a rather passive type of individual.

The internet, by contrast, is quite selective in the sense that powerful search engines allow users to access very specific content and information, respectively: Surveys among users show that the credibility of the internet is ranked rather high and environmental issues – in various languages – can certainly be accessed easily; moreover, in the field of the environment, the host side is rather varied since not only government information sites and scientific websites are accessible but also thousands of green non-governmental organization sites. The internet enjoys a rather high credibility among users Metzger (2007). The internet variable is also crucial from the perspective of knowledge diffusion and potential environmental networking and network effects, respectively.

The mobile phone could be a useful source of environmentally relevant information only if a high share of such phones offer mobile internet services which is indeed the case in high per capita countries. Statistics from ITU (2015) shows that the percentage of mobile phones with internet services in industrialized countries in 2014 was roughly three times as high as in developing countries; in the context of survey respondents in OECD countries, the two categories of mobile phone and internet certainly have a rather strong overlap. Mobile phone companies/telecommunication companies themselves often emphasize sustainability characteristics (e.g. Deutsche Telekom is using only electricity from renewable sources for the communication grid).

The role of friends for environmentally relevant information and concern, respectively, is somewhat unclear; obviously one may expect that all information obtained from friends will be assigned a high credibility, however, discussions with friends will often be centered mainly on non-environmental topics and issues.

These variables of interest are summarized in the below table (see Table 2):

**Table 2:** Overview of independent variables

Independent variables	Type	Description	Mean	SD
Internet	Ordinal 1–5	1 indicates that the respondent never uses internet to obtain information, 5 indicates that the respondent uses internet daily as an information source	2.85	1.80
Newspaper	Ordinal 1–5	1 indicates that the respondent never uses newspapers to obtain information, 5 indicates that the respondent uses newspapers daily as an information source	3.22	1.59
TV	Ordinal 1–5	1 indicates that the respondent never uses TV news to obtain information, 5 indicates that the respondent uses TV news daily as an information source	4.58	0.94
Radio	Ordinal 1–5	1 indicates that the respondent never uses radio news to obtain information, 5 indicates that the respondent uses radio news daily as an information source	3.44	1.65
Mobile Phone	Ordinal 1–5	1 indicates that the respondent never uses mobile phones to obtain information, 5 indicates that the respondent uses mobile phones daily as an information source		
Conversations	Ordinal 1–5	1 indicates that the respondent never uses conversations with friends or colleagues to obtain information about what is going on in this country and the world, 5 indicates that the respondent uses conversations with friends or colleagues daily as an information source	4.06	1.31

We also control for several socio-economic characteristics that are likely to have an impact on environmental concern. Among these characteristics are individual’s age, level of education, sex, offspring, satisfaction with financial situation, post-materialistic value orientation and environmental attitude.

From a theoretical perspective, an individual’s age is expected to have a negative impact on environmental concern. In addition, age might play a crucial role regarding the effect of information sources on environmental concern because younger people have easier to access to information about environmental issues than older people (Shen and Saijo 2007).

Regarding the individual’s level of education, one might assume that a better education is associated with more knowledge about many fields, including the state of the local, national and global environment – hence a positive sign is expected. Education also plays a crucial role for using any kind of information and news source relevant for environmental concern. For well-educated people, extracting relevant information from alternative conventional and digital information sources will be associated with rather low information costs since they have been trained during education to use alternative information sources purposefully – and hence marginal benefits from information sources might be higher for high-income groups than for low-income groups in which education levels are generally lower than in high-income groups.

Income might play two roles – a straightforward aspect that a higher income should go along with a higher demand for a clean environment (income elasticity of the demand for a clean environment is positive); at the same time, one may argue that a higher income allows individuals to have housing of high quality in the local environment which might reduce environmental concern. Having children has two important aspects – there is a need to take care of the child and hence the effectively tighter budget constraint of a family with children could reduce environmental concern; on the other hand, altruism in the normal family might reinforce environmental concern: The overall effect is unclear (Shen and Saijo 2007).

As regards the respondents’ sex, Schahn and Holzer (1990), Mohai (1992) and Hunter et al. (2004) suggest that women express greater concern for the environment than men. Since post-materialist values are much more apt at giving high priority to protecting the environment, Inglehart’s post-materialism index (Inglehart 1995) is also included and ranges from 0 to 5, with higher values indicating stronger post-materialistic value orientations. According to the value-belief-norm theory by Stern and Dietz (1994), Stern et al. (1995), Stern et al. (1999) and Stern (2000) assume a dominant role of values, norms and attitudes for environmental concern. Following this line of argumentation, we include responses to the question of whether looking after the environment, caring for nature and saving life resources are important for the respondent.

As individual-level responses are pooled across countries, unobservable cultural or geographic differences are considered by including country dummies. We also control for internet penetration.The control variables are summarized in the following table (see Table 3):

**Table 3:** Overview of control variables

Independent variables	Type	Description	Min	Max	Mean	SD
Age	Ordinal 1–9	1 indicates that the respondent is between 16 and 20 years old. 9 indicates that the respondent’s age is between 91 and 100	1	9	3,71	3,72
Female	Binary 0–1	1 if the respondent is female, 0 otherwise	0	1	0,53	0,49
Education	Ordinal 1–9	The respondent indicates the highest educational level that he or she have attained. 9 indicates university-level education with degree	1	9	5,77	2,42
Income	Ordinal 1–10	The respondent indicates how satisfied he or she is with the financial situation of his or her household. 10 indicates the highest satisfaction	1	10	5,96	2,47
Children	Binary 0–1	1 if the respondent has children, 0 otherwise	0	1	0,69	0,45
Post-materialism	Ordinal 1–4	Index based on 12 WVS questions, with higher values indicating stronger post-materialist value orientation	1	4	1,92	1,17
Eco_Person	Ordinal 1—6	The respondent indicates looking after the environment is important to this person, 6 indicates the highest agreement	4.50	1.26	1	6
Internet Use	Cont	Percentage of Individuals using the Internet	9	92.86	51.89	23.37

Since we will split the sample into high- and low-income sub-samples, it is appropriate to deliver some comparative statistics about some selected important variables in the corresponding sub-samples (see Table 4).

**Table 4:** Overview of independent variables – high-income vs. low-income

	High-income	Low-income
Variables	Mean	Mean
Affective	0.159	0.125
Conative	0.505	0.541
Behavioral	0.164	0.088
Internet	3.443	2.332
Newspaper	3.741	2.771
TV	4.618	4.551
Radio	3.670	3.250
Mobile Phone	3.211	2.975
Conversations	4.158	3.973
Age	47	39
Education	6.270	5.336
Fixed-broadband subscriptions (per 100 inhabitants)	21.074	6.375
Percentage of Individuals using the Internet	71.36	34.74
GDP per Capita	39,002.48	11,544.52

### Cross-section regressions

Since our corresponding dependent variables are binary, we apply common binary probit models on the pooled sample of individual responses. We consider the following baseline specification:

1 $$probit\left( {Env\_concern_{ij} = 1} \right) = \Phi \left( {\beta_{0} + \sum\limits_{k = 1}^{6} {\beta_{k} media_{k,ij} + \sum\limits_{k = 6}^{n} {\beta_{k} c_{k,ij} } + \gamma_{j} + u_{irj} } } \right)$$

$Env\_concern_{ij} \in \left\{ {0,1} \right\}$ are the binary responses of individuals *i* from country *j.* Since we are interested in the effect of different information sources on affective, conative and behavioral components of environmental concern, we will have three possible binary dependent variables. Different information sources which are supposed to have an effect on environmental concern are represented by $\sum\limits_{k = 1}^{5} {media_{k,ij} }$.$\sum\limits_{k = 8}^{t} {c_{k,ij} }$ represent control variables at the individual and country level,$\gamma_{j}$ are country dummy variables, and $u_{irj}$ are error terms.

### Results

This section presents the results of probit estimations based on the total sample and on different sub-samples. Subsequently, we look at the different pillars of environmental concern and focus on a model in two variants, namely, with and without country dummy. Since we are considering ICT variables as explanatory variables, one should be careful in the interpretation of the results since ICT is also raising per capita income (another explanatory variable), but at the bottom line we show a distinct impact of digital variables and the internet, respectively.

Table 5 reports the marginal effects of the explanatory variables on the probability that the respondent is environmentally concerned. The model specifications (1a) and (1b) refer to the affective dimension of the environmental concern. (1b) differs from (1a) through the inclusion of country dummies and the exclusion of internet penetration. We apply the same procedure for other specifications. (2a) and (2b) refer to the conative dimension and (3a) and (3b) refer to the behavioral dimension of environmental concern, respectively.

**Table 5:** Probit estimation results based on full sample – marginal effects

Explanatory variables	Affective (1a)	Affective (1b)	Conative (2a)	Conative (2b)	Active (3a)	Active (3b)
Internet	0.0001 (0.001)	0.004*** (0.001)	0.001 (0.002)	0.004** (0.002)	0.010*** (0.001)	0.009*** (0.001)
Newspaper	0.004*** (0.001)	-0.003** (0.001)	0.007*** (0.002)	-0.004** (0.002)	0.013*** (0.001)	0.009*** (0.001)
TV	0.010*** (0.002)	0.002 (0.002)	-0.001 (0.003)	-0.008** (0.003)	-0.014*** (0.001)	-0.008*** (0.001)
Radio	-0.005*** (0.001)	0.001 (0.001)	0.002 (0.001)	0.003** (0.002)	0.008*** (0.001)	0.006*** (0.001)
Mobile phones	-0.002** (0.001)	-0.003*** (0.001)	-0.004** (0.001)	-0.008*** (0.001)	0.005*** (0.001)	0.005*** (0.001)
Conversation	-0.005*** (0.001)	-0.001 (0.001)	-0.003* (0.002)	0.001 (0.002)	-0.001 (0.001)	0.0002 (0.001)
Female	-0.014*** (0.003)	-0.014*** (0.003)	0.009* (0.005)	0.010** (0.005)	-0.003 (0.003)	-0.001 (0.002)
Age	-0.0002 (0.001)	-0.002** (0.001)	-0.001 (0.002)	0.001 (0.002)	0.006*** (0.001)	0.008*** (0.001)
Income	- 0.0004 (0.001)	0.001** (0.001)	0.006*** (0.001)	0.004*** (0.001)	0.007*** (0.001)	0.005*** (0.001)
Education	0.009*** (0.001)	0.003*** (0.001)	0.011*** (0.001)	0.012*** (0.001)	0.006*** (0.001)	0.007*** (0.001)
Children	-0.002** (0.001)	-0.003 (0.004)	0.001 (0.001)	-0.007 (0.006)	0.005*** (0.001)	0.006* (0.003)
Post-materialism	0.001 (0.001)	0.002* (0.001)	0.045*** (0.002)	0.042*** (0.002)	0.022*** (0.001)	0.016*** (0.001)
Eco_Person	0.017 *** (0.001)	0.022*** (0.001)	0.063*** (0.002)	0.063*** (0.002)	0.027*** (0.001)	0.026*** (0.001)
Internet Use	0.001*** (0.0001)		-0.0002 (0.0001)		0.001*** (0.0001)	
Country dummy	No	Yes	No	Yes	No	Yes
Prob > chi2	0.000	0.000	0.000	0.000	0.000	0.000
Pseudo R2	0.022	0.093	0.031	0.0670	0.0923	0.127
Number observations	55,171	55,171	51,687	51,687	55,185	55,185

Marginal effects calculated at the means of the variables reported. Robust standard errors in parentheses

*p < 0,l; **p < 0,05; ***p < 0,01

Based on the results there is clear evidence for the H1 hypothesis. Internet has a positive significant effect on the probability that the corresponding respondent is environmentally concerned for all dimensions if we include country dummies. Internet has a significant negative effect on the affective dimension of environmental concern. However, internet also has a significant positive effect on the affective dimension after including country dummies. One can also see the positive effect by considering the percentage of individuals using the internet which has a significant positive effect on the affective and behavioral dimensions of environmental concern.

As regards the second hypothesis, H2, one can see that by including the country dummies, almost all information sources are significant for conative and behavioral dimensions of environmental concern. However, only internet, newspaper and mobile phones significantly influence the affective dimension of environmental concern. Thus, we can accept the H2 hypothesis for the full sample.

Regarding other information sources, using newspapers has a negative effect on the affective and the conative dimensions of environmental concern. However, newspaper has a positive impact on the behavioral dimension of the environmental concern. Using radio as an information source has a significant positive effect on the affective and the conative dimensions of environmental concern. Mobile phone use has a negative impact on the affective and conative dimensions of respondents’ environmental concern. However, it has a significant effect on the behavioral dimension of environmental concern. Talking with friends and colleagues has no effect on environmental concern. Only for the conative dimension of environmental concern is there a significant positive effect of conversations as an information source. But this effect disappears after controlling for country dummies.

Table 6 reports the results of probit estimations based on the high-income sub-sample and replicates some key results from the full sample.

**Table 6:** Probit estimation results based on high-income sub-sample – marginal effects

Explanatory variables	Affective	Affective	Conative	Conative	Active	Active
Internet	-0.007*** (0.002)	0.004** (0.002)	0.002 (0.002)	0.006** (0.003)	0.014*** (0.002)	0.009*** (0.002)
Newspaper	-0.002 (0.002)	-0.006*** (0.002)	-0.009*** (0.003)	-0.009*** (0.003)	0.016*** (0.002)	0.013*** (0.002)
TV	0.023*** (0.003)	0.004 (0.003)	-0.006 (0.004)	-0.017*** (0.004)	-0.012*** (0.003)	-0.007** (0.003)
Radio	-0.010*** (0.002)	0.0004 (0.002)	0.010*** (0.002)	0.011*** (0.003)	0.007*** (0.002)	0.007*** (0.002)
Mobile phones	-0.003** (0.001)	-0.003* (0.002)	-0.007*** (0.002)	-0.007*** (0.002)	0.004** (0.001)	0.005*** (0.001)
Conversation	0.004 (0.002)	0.001 (0.002)	0.009** (0.003)	0.003 (0.003)	-0.002 (0.002)	0.002 (0.002)
Female	-0.025*** (0.005)	-0.023*** (0.005)	0.001 (0.007)	0.004 (0.007)	0.011*** (0.005)	0.011** (0.005)
Age	-0.006 (0.002)	-0.003 (0.002)	-0.009** (0.002)	-0.001 (0.003)	0.011*** (0.002)	0.010*** (0.002)
Income	-0.004*** (0.001)	0.002* (0.001)	0.001 (0.002)	0.0003 (0.002)	0.008*** (0.001)	0.005*** (0.001)
Education	0.012*** (0.001)	0.003** (0.001)	0.011*** (0.002)	0.014*** (0.002)	0.010*** (0.001)	0.012*** (0.001)
Children	-0.002 (0.002)	-0.001 (0.006)	0.006** (0.002)	-0.001 (0.009)	-0.003* (0.002)	0.004 (0.006)
Post-materialism	-0.009*** (0.002)	-0.003 (0.002)	0.066*** (0.003)	0.063*** (0.003)	0.027*** (0.002)	0.024*** (0.002)
Eco_Person	0.028*** (0.002)	0.035*** (0.002)	0.089*** (0.003)	0.091*** (0.003)	0.047*** (0.002)	0.048*** (0.002)
Internet use	0.004*** (0.0002)		-0.0004* (0.0002)		0.004*** (0.0001)	
Country dummy	No	Yes	No	Yes	No	Yes
Prob > chi2	0.000	0.000	0.000	0.000	0.000	0.000
Pseudo R2	0.031	0.099	0.055	0.080	0.101	0.131
Number observations	24,227	24,227	22,212	22,212	24,182	24,182

Marginal effects calculated at the means of the variables reported. Robust standard errors in parentheses

*p < 0,l; **p < 0,05; ***p < 0,01

For the high-income sub-sample there is also evidence for the H1 hypothesis since internet as an information source is always positive and significant after including country dummies. Without including country dummies internet has a negative effect on the affective dimension of environmental concern.

As regards the H2 hypothesis (the higher the individual commitment for the environment is, the broader the information basis used will be), almost all information sources have a positive significant effect on the conative and behavioral dimensions of environmental concerns. This does not apply for the affective dimension. Thus, we can accept the H2 hypothesis for the high-income sub-sample. Consequently, cooperation amongst OECD countries should be easier – not only because of similarities in terms of per capita income but because similar digital information intensity will also be observed so that environmentally-relevant news and information items are shared rather often—more so than between high income countries and low income countries.

Regarding other information sources, the impact of newspapers as an information source is negative for the affective and conative dimensions, but significantly positive for the behavioral dimension. Radio has a negative impact on the affective dimension, but a positive impact on the conative and behavioral dimensions of environmental concern. Mobile phone use has a negative impact on the affective and conative dimensions of environmental concern; however, the impact on the behavioral dimension of environmental concern – if country dummies are included – is positive. Talking with friends and colleagues as an information source has a positive impact only on the conative dimension of environmental concern (and if not controlled for country dummies).

As regards the percentage of individuals using the internet, it has a positive impact on the affective and behavioral dimensions of environmental concern. At the bottom line, one may argue that internet use is very important since internet is a crucial source of information with respect to environmental concern (this holds for all sub-pillars of concern) while the internet penetration variable for the respective country also has a positive impact on both conative and behavioral dimensions. Table 7 finally provides the results of probit estimations for the low-income sub-sample.

**Table 7:** Probit estimation results based on low-income sub-sample – marginal effects

Explanatory variables	Affective	Affective	Conative	Conative	Active	Active
Internet	0.005*** (0.001)	0.003** (0.001)	0.001 (0.002)	0.002 (0.002)	0.006*** (0.001)	0.009*** (0.001)
Newspaper	0.007*** (0.001)	-0.001 (0.001)	0.019*** (0.002)	0.002 (0.002)	0.011*** (0.001)	0.008*** (0.001)
TV	0.005 (0.002)	0.0003 (0.002)	0.002 (0.003)	-0.001 (0.003)	-0.012*** (0.002)	-0.008*** (0.002)
Radio	-0.002* (0.001)	0.0004 (0.001)	-0.002 (0.002)	-0.002 (0.002)	0.006*** (0.001)	0.005*** (0.001)
Mobile phones	-0.001 (0.001)	-0.003** (0.001)	0.00001 (0.002)	-0.008*** (0.002)	0.007*** (0.001)	0.005*** (0.001)
Conversation	-0.008*** (0.001)	-0.002 (0.001)	-0.008*** (0.002)	0.0002 (0.002)	-0.001 (0.001)	-0.001 (0.001)
Female	-0.005* (0.004)	-0.008** (0.003)	0.014* (0.006)	0.013** (0.006)	-0.013*** (0.003)	-0.010*** (0.003)
Age	0.007 (0.001)	-0.002 (0.001)	0.011** (0.002)	0.004* (0.002)	0.001 (0.001)	0.005*** (0.001)
Income	0.001* (0.001)	0.001* (0.001)	0.010*** (0.001)	0.006*** (0.001)	0.005*** (0.001)	0.004*** (0.001)
Education	0.006*** (0.001)	0.003*** (0.001)	0.009*** (0.001)	0.009*** (0.002)	0.004*** (0.001)	0.004*** (0.001)
Children	0.004** (0.001)	-0.004 (0.004)	-0.004** (0.002)	-0.012 (0.008)	0.006*** (0.001)	0.003*** (0.004)
Postmaterialism	0.009*** (0.002)	0.006*** (0.002)	0.031*** (0.003)	0.023*** (0.003)	0.016*** (0.001)	0.009*** (0.001)
Eco_Person	0.012*** (0.002)	0.014*** (0.001)	0.046*** (0.002)	0.044*** (0.002)	0.015*** (0.001)	0.011*** (0.001)
Internet use	0.001*** (0.0001)		0.001*** (0.0002)		0.0003** (0.0001)	
Country dummy	No	Yes	No	Yes	No	Yes
Prob > chi2	0.000	0.000	0.000	0.000	0.000	0.000
Pseudo R2	0.023	0.0848	0.027	0.064	0.0613	0.093
Number observations	30,944	30,944	29,475	29,475	31,003	31,003

Marginal effects calculated at the means of the variables reported. Robust standard errors in parentheses

*p < 0,l; **p < 0,05; ***p < 0,01

As regards the H1 hypothesis, it can be accepted only for the affective and behavioral dimensions of environmental concern. Internet is not significant for the conative dimension. It is interesting that across all model specifications without country dummies, newspaper has a positive impact – possibly reflecting the fact that newspapers’ coverage of major international environmental conferences (e.g. COP 21 or COP25 or the publication of major environmental reports) or of public actions of Greenpeace and other environmental groups gives relevant information for the pro-environmental attitude in low-income countries (and weak evidence exists also for the conative dimension; this rather weak role of newspaper – compared to high-income countries—for the two basic dimensions of concern might reflect the fact that illiteracy in many developing countries could still play some role for information sourcing).

The H2 hypothesis can also only partly be accepted since information sources seem to have a restricted effect on the conative component of the environmental concern. The H3 hypothesis states that the use of mobile phones as an information source influences environmental concern in the low-income sub-sample. This H3 hypothesis can be accepted since the use of mobile phones is the only variable which is significant for all dimensions of environmental concern for the low-income sub-sample.

It is also notable that using newspaper as an information source has a positive effect on all sub-pillars of environmental concern in the setup without country dummies. However, after including country dummies, newspaper use has a positive impact only on the behavioral dimension. Radio is strongly positively significant with respect to action. Conversation has a negative impact on the conative and behavioral dimensions – if not controlled for country dummies. After including country dummies, talking with friends and colleagues is not significant at all. As regards the percentage of individuals using the internet, it has a positive impact on all dimensions of environmental concern. This again reinforces the view that digital information plays a key role for environmental concern.

## Conclusion and policy implications

Based on the results of the probit estimations, there is clear evidence for the H1 and H2 hypotheses for both the full sample and the high-income sub-sample. Internet has a positive significant effect for all dimensions of environmental concern after the inclusion of country dummies. There is also empirical evidence that weak levels of environmental concern are linked to a rather narrow information status; the higher the individual commitment to the environment is, the broader the information basis used will be.

As regards the low-income sub-sample, the internet is not significant as an information source for the conative dimension of environmental concern. The H2 hypothesis can also only partly be accepted since information sources seem to have a restricted effect on the conative component of environmental concern. The H3 hypothesis can be accepted since the use of mobile phones is the only variable which is significant for all dimensions of environmental concern for the low-income sub-sample.

However, it should be noted that as our analysis is based on cross sectional data, the results need to be interpreted as correlations. This caveat does not mean that the basic considerations and conclusions are not adequate. One key aspect is the general economic perspective that the expansion of information and communication technology – including the internet – has a positive role with regard to economic growth, more so in OECD countries than in developing countries (World Bank 2013, 2020). Internet expansion thus may be expected to contribute to higher per capita income, but the more important aspects with respect to environmental concern is the information-enhancing quality of the internet.

The relevant sources of information differ across income groups: mainly rich OECD member countries and other relatively poor countries. As developing countries may be assumed to catch-up with OECD countries, not only in terms of per capita income but also in terms of internet use/internet density, the prospects for international cooperation between developed and developing countries should be improving over time. With more similar per capita income, the typical interests of the median voters in the two country groups should become more similar and this could reinforce the opportunities for international cooperation. Since mobile phone density in developing countries – with mobile phones having internet access on a large scale since about 2010 (ITU 2018) – is catching up quickly with that of OECD countries, and since digital communication convergence dynamics are clearly progressing faster than per capita income convergence in a Global North–South perspective, the opportunities for the creation of broader North–South information and knowledge networks are enhanced through digital communication.

As regards the age variable: there could be a declining role of environmental concern in the long run in OECD countries as the ageing of society will play a crucial role in coming decades. In developing countries, ageing will be a less important phenomenon from a demographic perspective, but the sign is opposite to that in high-income countries. To the extent that there is a broad international consensus that certain environmental problems – e.g. climate protection (as emphasized by COP 21 and previous conferences) – require joint action, the activities of the World Bank and ITU in encouraging digital modernization in developing countries could critically contribute to more political legitimacy of such activities since more people will share deeper environmental concern if internet sources become more widely available. Thus, the internet could also be useful in helping to provide a global public good such as climate protection. The fact that the US under President Trump has withdrawn from the Paris Climate Agreement raises new interesting questions to the extent that Trump’s massive twitter campaigns in all major policy fields seems to have an influence on climate skeptical groups in the US (Schuldt et al. 2017; Sojung and Cooke 2018). Digital political anti-environmental leadership could obviously undermine the role of digital communication channels in OECD countries as an impulse for reinforcing environmental concern and the popular interest in better climate change policies. Future research could also look into the apparently ongoing environmental party polarization tendencies in the US after 2000 (Karol 2018).

We have shown important findings for high-income countries and low-income countries, respectively. Some of the findings are relevant with respect to the particular question of changing opportunities for green international cooperation: The assumption is that if the environmental preferences are more similar across countries in the medium term, the prospects for international environmental cooperation are improving; and as the survey results, in combination with some other dynamics, indeed have such an implication, there is no need to be generally skeptical with respect to solving environmental problems. One may argue that there is a medium time range of about 2020–2040 (or 2050) after which the greying of the population might undermine the willingness to pay in many countries and this, after a critical point in time, will make international cooperation more difficult.

With poor countries catching-up in both economic terms and education levels, the global demand for environmental quality and the environmental concern, respectively, will increase. While it is not really known to what extent the low-income countries included in the WVS 2010–14 are representative of the developing world, one may argue that there is a considerable likelihood for the population in catching-up countries to become more pro-environmentally oriented. In some cases, there is external pressure from outside and trade interests, respectively; e.g., the case of fishing standards in Thailand and other ASEAN countries on which the EU’s fisheries policy has an indirect influence (Tavornas and Cheeppensook 2019).

Since 60% of the global population lives in Asia, Asian economic and education dynamics are of particular interest; as Wößmann et al. (2015) have shown, Asian countries’ effective progress in improving education levels is considerable and actually higher than in Latin American countries. From this perspective, the medium-term outlook for more international environmental cooperation is favorable as economic catching-up processes in both Asia, Latin America and Africa are continuing. Moreover, the ITU (2015) expects that developing countries will catch up in term of internet density by 2020 or a few years later. Both the falling relative prices of ICT products and services (Welfens and Perret 2014) and digital network effects should contribute to stimulating a strong digital catching-up process of developing countries. The increasing global internet intensity in turn lets one expect that willingness to pay in developing countries will increase beyond the positive income elasticity effect of the demand for a clean environment.

As regards policy implications, one can find differences between the high-income group and the low-income group. These differences could indirectly reflect some form of environmental Kuznets curve so that beyond a critical per capita income peak, the green willingness to act is more clearly related to certain key variables than is the case in a low-income group of countries. One cannot rule out that in a high-income country group this finding is related to the typically more sophisticated communication and information infrastructure found in high per capita income countries: in a high communication society the creation of new advanced social norms might be much easier than in a poor society with weak communication links. It is well-known in diffusion models of social behavior that the frequency of personal interaction or information exchange shapes the speed of diffusion and thus the ability to create a new social norm in a rather short period. One may indeed assume that societies with rather low diffusion rates of information/products will find it difficult to establish any innovative standard easily; if diffusion is very slow, the existing sets of behavior will prevail, even if the attitudes of the younger age cohorts might have changed. Obviously, education is a rather relevant variable.

However, one should not rule out that better education is not only relevant in the sense that individual information processing costs are rather low, but one may also anticipate that a rather high education standard of the majority of society will bring about a more intensive competition of new ideas so that the opportunities for new green standards is enhanced from the supply-side in the political and social process: Establishing new ambitious standards is easier if there is a broader variety of advanced standards that enter the social race up to a new environmental standard.

From a policy perspective, there are three key challenges to be considered:

- how can economic convergence be reinforced for rather poor societies since if a rather poor country joins the group of high per capita income countries the links between attitudes and actions are shaped by a new regime;
- how can government encourage more ambitious attitudes, e.g. by setting good examples in the public sphere or by pushing more for internalization of negative external effects from sectors with high pollution or high GHG emissions; if the sectoral – or regional – progress achieved thereby is supported by political public relation campaigns at the national level a higher national standard could be developed;
- to the extent that the subsidization of the expansion of digital networks and information & communication technology, respectively, stimulates the diffusion of more advanced green standards, government’s Digital Policy could play a crucial role in many countries.

A major finding is that depending on the level of commitment to environmental protection the range of information sources is clearly broadened. This suggests that individual willingness to pay for the environment – here, donating to a green foundation or action group – is very much related to reliable information sources and possibly also to digital networking opportunities. There is, of course, a serious caveat as regards the role of digital networking and pro-sustainability attitudes: In principle, digital social media could be used as much for promoting pro-environmental attitudes and green policy approaches as it could be used by powerful groups interested in continued fossil fuel-based energy production to fend off broader sustainability policy: While a large share of the digital services offering online appear to be free to use for the general public, one should not overlook that the cost of running digital platforms and social networks are considerable – wealthy individuals or firms interested in achieving certain goals could try to manipulate the public in favor of minimal climate change policy (one can consider the case of voter manipulation in the US in the presidential campaign of 2016 and the role of Cambridge Analytica, respectively).

With the OECD green growth initiatives, on the one hand, and the leading telecommunication firms, on the other, as drivers of energy-efficient networks and digital services – often based on the use of renewable energy – the digital sphere is likely to play an increasing role for environmentally relevant information and networking activities. As regards networking activities, one may consider interdependent actions among firms or among households. The Smarter 2020 initiative (later renamed Smarter 2030) is an example from leading telecommunication network operators to create green digital networked activities on the ICT supply-side (GeSI 2012; GeSI 2015); and indeed, one can also find green ICT dynamics for Germany that are partly related to the supply-side of the ICT sector and partly related to digital information diffusion and green networking in the field of sustainable consumption and investment (Welfens and Lutz 2012). Linked or interdependent individual actions can play a role in economic and ecological fields for various reasons. As regards firms in a given sector, one may wonder about the supply-side structure, namely, if there is oligopolistic interdependence and hence some form of ‘follow the leader’ behavior. The role of green information & communication technology for OECD countries has been emphasized in many contributions; in a regional context, e-waste recycling behavior and attitudes in China have been analyzed (Wang et al. 2011). 

With the shock of the coronavirus pandemic shocks and the Corona World Recession (Weflens 2020), there has been strong pressure in many countries to expand the digital sector – for example, in the context of digital home office work, telecommunications apps and more digital teaching and e-learning offerings. This international impulse for the growth of the ICT sector could reinforce the digital communication channels relevant for green progress in very many countries in most regions of the world economy. It is also noteworthy that the expansion of ICT-intensive production raises the demand for skilled labor and a better educated workforce is likely to be more interested in a cleaner environment than previous generations. However, the strong rise of unemployment in most OECD countries and many developing countries in 2020 might also create a conflict of interest between green progress and job security in many countries worldwide for a transition period.

In the long run, it is crucial to achieve convergence of both climate-policy attitudes and per capita income in individual countries – to the extent that there is no significant correlation between the two variables – in the field of global climate policy, as otherwise large international discrepancies in CO2 tax rates and CO2 emission certificate prices (see IMF 2019) would continue so that considerable efficiency losses in climate policy would be realized.

For future research, there are a number of topics and issues to be considered. For example, it is unclear how ageing really affects the green action variable since in an ageing society the median age will rise. At the same time, a rising average life expectancy implies that the benefits from green action/environmental progress in the current period t will generate an extra temporal benefit for the additional (marginal) years of additional life expectancy.

It will also be interesting to further explore the green ICT dynamics both among firms in that sector and in the ICT-using sectors. The Corona shock of 2020/2021 has reinforced digital expansion and thus could contribute to faster information about sustainability worldwide. To some extent ICT expansion means to replace traditional high resource intensity and high energy intensity activities by a less environmentally-intensive digital production and consumption format. The number of countries included in the World Value Survey should be increased in the long run so that the data series will become broader and unbalanced panel data analysis will become possible. A key suggestion for the questions posed is not only to ask about the attitude and action of the respective person, but also to formulate some questions about the perceived view of the majority on certain issues and about the role of digital networking as well as the relative reliability of the internet for different fields (e.g. economic topics, environmental topics, foreign policy issues); moreover, it would be interesting to get a view as to what extent international climate burden sharing is viewed as fair or as to whether or not communal green activities (e.g., recycling) are considered as key to green environmental learning-by-doing at a community level. Thus, there is a broad and rich research agenda for the future.
